# Pathophysiology of myelin oligodendrocyte glycoprotein antibody disease

**DOI:** 10.3389/fneur.2023.1137998

**Published:** 2023-02-28

**Authors:** Osman Corbali, Tanuja Chitnis

**Affiliations:** ^1^Harvard Medical School, Boston, MA, United States; ^2^Department of Neurology, Brigham and Women's Hospital, Ann Romney Center for Neurologic Diseases, Boston, MA, United States

**Keywords:** MOGAD, T cells, MOG (myelin oligodendrocyte glycoprotein), blood brain barrier (BBB), MOG-IgG, autoantibodies, pathophysiology-contemporary knowledge

## Abstract

Myelin Oligodendrocyte Glycoprotein Antibody Disease (MOGAD) is a spectrum of diseases, including optic neuritis, transverse myelitis, acute disseminated encephalomyelitis, and cerebral cortical encephalitis. In addition to distinct clinical, radiological, and immunological features, the infectious prodrome is more commonly reported in MOGAD (37–70%) than NMOSD (15–35%). Interestingly, pediatric MOGAD is not more aggressive than adult-onset MOGAD, unlike in multiple sclerosis (MS), where annualized relapse rates are three times higher in pediatric-onset MS. MOGAD pathophysiology is driven by acute attacks during which T cells and MOG antibodies cross blood brain barrier (BBB). MOGAD lesions show a perivenous confluent pattern around the small veins, lacking the radiological central vein sign. Initial activation of T cells in the periphery is followed by reactivation in the subarachnoid/perivascular spaces by MOG-laden antigen-presenting cells and inflammatory CSF milieu, which enables T cells to infiltrate CNS parenchyma. CD4+ T cells, unlike CD8+ T cells in MS, are the dominant T cell type found in lesion histology. Granulocytes, macrophages/microglia, and activated complement are also found in the lesions, which could contribute to demyelination during acute relapses. MOG antibodies potentially contribute to pathology by opsonizing MOG, complement activation, and antibody-dependent cellular cytotoxicity. Stimulation of peripheral MOG-specific B cells through TLR stimulation or T follicular helper cells might help differentiate MOG antibody-producing plasma cells in the peripheral blood. Neuroinflammatory biomarkers (such as MBP, sNFL, GFAP, Tau) in MOGAD support that most axonal damage happens in the initial attack, whereas relapses are associated with increased myelin damage.

## Introduction

MOG is a transmembrane protein found on the outer surface of the central nervous system myelin and a marker of mature oligodendrocytes (1, 2). It constitutes only a small portion of the myelin (0.05%), and its possible roles include cell adhesion, microtubule stability, and receptor function (1, 3, 4).

High titers of autoantibodies targeting MOG are identified in various demyelinating diseases, including optic neuritis, transverse myelitis, acute disseminated encephalomyelitis (ADEM), and cerebral cortical encephalitis. These are now recognized as a spectrum of diseases associated with MOG antibodies, MOGAD (5). Despite heterogeneous presentation and clinical overlap between MOGAD, multiple sclerosis (MS), and neuromyelitis optica spectrum disease (AQP4-IgG+, NMOSD), distinctive radiologic, pathological, lab, and clinical features of MOGAD have been identified (Table 1), and most recently an international MOGAD diagnostical criteria has been proposed (63).

**Table 1:** Distinctive features of MOGAD.

	**MOGAD**	**NMOSD**	**MS**
**Clinical findings**			
Presentation at onset	Optic neuritis	Optic neuritis	Optic neuritis
	Transverse myelitis	Transverse myelitis	Transverse myelitis
	Brainstem demyelination	Brainstem demyelination	Brainstem demyelination
	ADEM	Area postrema syndrome	Internuclear ophthalmoplegia
	Cerebral cortical encephalitis		
Infectious Prodrome (6–14)	37.5–70% (at least once)	15–35% (at least once)	27–48% (of all relapses)
Course (6, 15–17)	Monophasic (40–50%)	Monophasic (10%)	Relapsing Remitting (90%)
	Relapsing (50–55%)	Relapsing (90%)	Secondary Progressive (half of relapsing remitting patients develop secondary progressive disease)
			Primary Progressive/Relapsing Progressive (10%)
Annualized Relapse Rate (excluding the first attack) (5, 18, 19) [Overall: treated and untreated]	0.23 (pediatric, overall) 0.35 (adult, overall)	0.91 (adult, untreated) 0.18 (adult, treated)	1.13 (pediatric, overall) 0.40 (adult, overall)
Progression independent of relapse activity (PIRA) (20, 21)	No	No	Yes
**Radiology**			
Optic nerve (22, 23)	Bilateral	Bilateral	Unilateral
	Lengthier	Lengthier	Shorter
	Anterior optic pathway involvement	Posterior optic pathway involvement	
	ON head swelling		
	Perineural sheath enhancement		
Spinal cord (22, 24, 25)	LETM	LETM	Shorter lesions
	Multiple lesions	Central cord	Multiple lesions
	H-sign (gray matter restricted lesion)		Dorsal/lateral lesions
	Central cord		Conus medullaris
	Conus medullaris		
Brain (22, 26, 27)	Less supratentorial lesions	Less supratentorial lesions	More supratentorial lesions
	ADEM-like lesions	Diencephalon (i.e., hypothalamus and thalamus)	Cortical/juxtacortical lesions
		Dorsal midbrain (i.e., area postrema)	
Contrast Enhancement rate within 4 weeks of the attack (Indication of BBB damage) (23, 24, 28)	ON (94%) Myelitis (26–70%)	ON (100%) Myelitis (78%)	ON (75%) Myelitis (75%)
Leptomeningeal enhancement (29–31)	33% (Pediatric)	6%	21% (1.5 or 3 T field)
	6% (Adult)		79% (7 T field)
Central Vein sign (average CVS+ rate) (22)	≈10%	< 10%	>40%
Slowly expanding lesions (25, 32)	No	Not studied	Yes
Paramagnetic rim lesions (32–34)	Not studied	Rare	Yes (due to iron laden microglia/macrophage)
**Lab**			
Serum ab test (35–37)	High Mog-IgG titer	High AQP4-IgG titer	Low Mog-IgG titer possible
Longitudinal ab testing (38–40)	Some patients become seronegative	Rarely becomes seronegative	NA
Oligoclonal bands (30, 41–46)	5–13%	10–16%	95%
Increased Qalb rate (% higher than the age normal; indication of Blood-CSF barrier damage) (10, 45–48)	32–35% (all patients)	ON (15%) myelitis (64%)	25% (all patients)
CSF pleocytosis (during acute attack) (42, 45, 47)	Optic neuritis: 34% Myelitis: 85% Brain/Brainstem: 60%	Optic neuritis: 24% Myelitis: 65%	50%
**Pathology**			
White matter lesions (49)	Perivenous confluent around small veins	Perivenous confluent/focal	Focal lesions around large veins
	Deep white matter	High AQP4 expressing regions (hypothalamus, area postrema), but also supratentorial	Periventricular
	Chronic active lesions absent		Chronic active and slowly expanding lesions
		Iron rim lesions absent		Iron rim lesions present
Cortical lesions (50–54)	Perivenous confluent intracortical demyelination	No cortical demyelination	Band-like subpial demyelination underneath the meningeal inflammation
		Neuronal loss in cortical layers II-IV	Ectopic meningeal lymphoid follicles
Dominant T cell type (50, 55–57)	CD4	Increased activated CD4 T cells (OX40+) reported	CD8
Activated complement deposition (49, 50, 58)	Present	Present	Present
Astrocytes (49, 50, 58–61)	Relative sparing	Pronounced loss	Activated and contribute to inflammation
	Normal GFAP (CSF)	Increased GFAP (CSF)	Increased GFAP (CSF and serum) in progressive MS
Oligodendrocytes (49, 50, 58, 62)	Variable loss	Variable loss	Variable loss (Type III demyelination)
	Preserved progenitor cells		

Autoimmunity in MOGAD starts at the periphery by activation of T cells and production of autoantibodies and eventually transfer of these immune mediators into the CNS (5, 38). How humoral immunity and cellular immunity cooperate in MOGAD pathogenesis is an intriguing subject. This review will highlight major distinctive clinical, radiological, and histopathological features of MOGAD and summarize how different immune compartments contribute disease pathogenesis. For this purpose, we will use evidence from human and animal studies such as MOG-induced experimental autoimmune encephalomyelitis (EAE) models, as some of these models closely resemble MOGAD compared to MS due to relapsing and remitting course (vs. progressive disease) and MOG as the autoimmune trigger (64–66).

## Distinctive features of MOGAD

Distinctive clinical, radiological, and histopathological features and laboratory findings of MOGAD are summarized below (Table 1).

### Clinical findings

MOGAD most commonly presents as bilateral optic neuritis, transverse myelitis, ADEM, or, less commonly, cerebral cortical encephalitis. Brainstem demyelination could also occur in MOGAD; however, area postrema syndrome or internuclear ophthalmoplegia is associated with NMOSD or MS, respectively (5, 67).

The monophasic course is more common in MOGAD (40–50%), and the remaining half of the MOGAD cases experience a relapsing course, which is associated with persistent high titers of MOG-IgG (15, 16). In contrast, most NMOSD patients (90%) have a relapsing course (6). In MS, most patients first experience a relapsing-remitting phase (90%), half of which develop secondary progressive disease (17). Progression in MOGAD or NMOSD is relapse dependent; however, progression independent of relapse activity is well-established with MS (20, 21).

Pediatric MOGAD is not more aggressive than adult-onset MOGAD. The overall annualized relapse rate (ARR) in MOGAD, excluding the first attack, is 0.23 in pediatric and 0.35 in adult MOGAD patients (5). This is different from MS, where pediatric MS patients display a more inflammatory phenotype and therefore have higher ARR than adult-onset MS patients, with an ARR of 1.13 in pediatric-onset vs. 0.40 in adult-onset MS (18).

An infectious prodrome (at least once during the disease course) is commonly reported with MOGAD, varying from 37 to 70% (7–10). MOG-IgG+ optic neuritis patients had 37% and 67% preceding infection in two series (3/8 and 6/9) (7, 8). In MOG-IgG+ ADEM patients preceding infection was present in 70% (12/17, including three patients with vaccinations) (9). Jarius et al. reported an infectious prodrome in 40% (15/37) of MOGAD patients, which included mostly optic neuritis or myelitis patients but also some ADEM or cerebellitis cases (10). In NMOSD, the infectious prodrome is reported in 15–35% (6, 11, 12). Earlier studies with multiple sclerosis showed that 27–48% of all MS relapses were associated with infections if patients followed longitudinally (13, 14).

### Radiological findings

**Figure 1:**
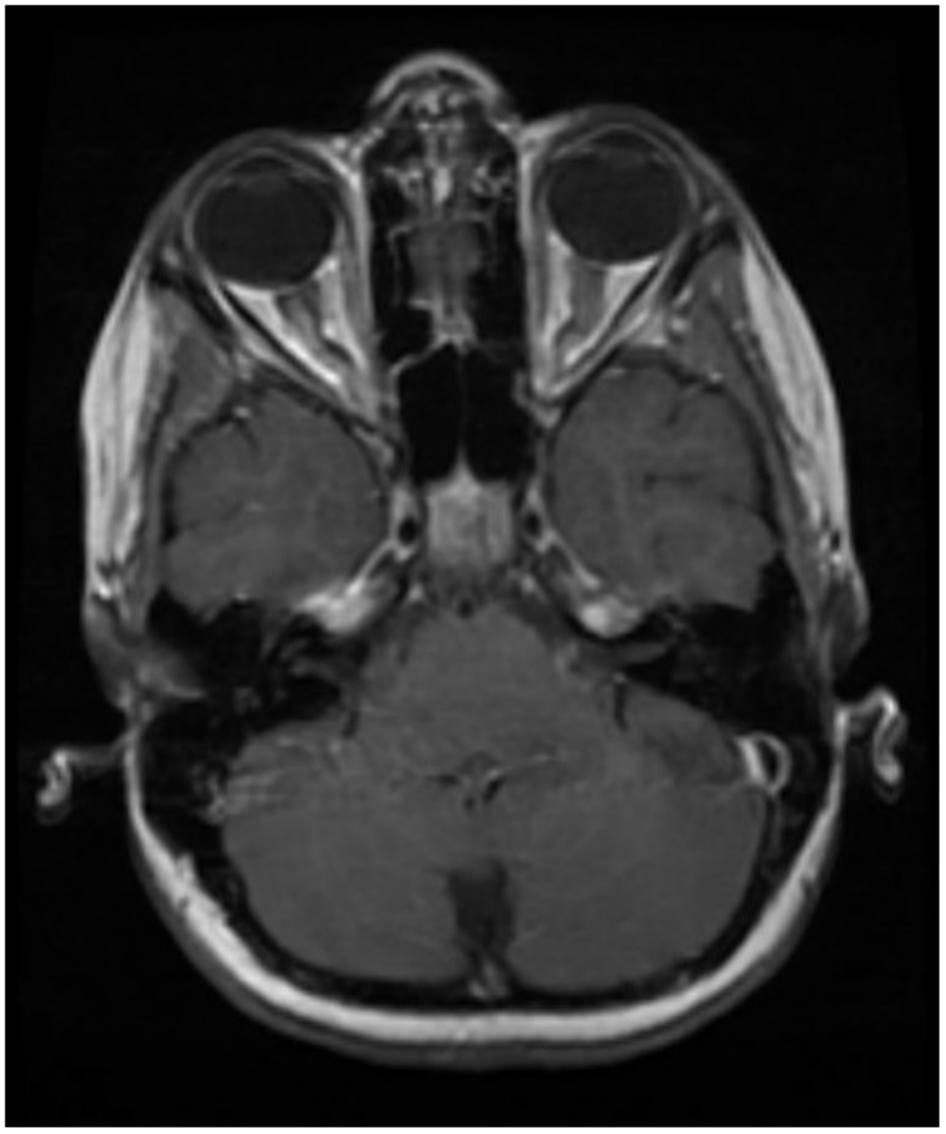
Typical optic nerve involvement in MOGAD. Bilateral and longer lesions involving anterior parts of the optic nerve.

Optic neuritis is bilateral and lengthier (compared to MS), and the anterior optic pathway is more commonly involved (vs. posterior in NMOSD) in MOGAD (Figure 1) (22, 23, 68). Optic nerve head swelling and perineural sheath enhancement are other typical features of MOGAD, indicating increased blood—optic nerve barrier breakdown (22, 68).

Spinal cord involvement is seen as longitudinal extensive transverse myelitis (LETM) in MOGAD and NMOSD (22, 24, 25). Multiple spinal cord lesions are frequent in MOGAD and MS (both >60%) (24). Gray matter restricted (H sign) or central cord involvement is a typical appearance of MOGAD lesions on axial MRI (24, 25). On the other hand, shorter and dorsal/lateral spinal cord lesions are more typical of MS (25). Conus medullaris involvement is seen in MOGAD or MS (22).

Typical brain involvement in MOGAD is ADEM-like fluffy lesions (25). The number of supratentorial lesions is fewer in MOGAD and NMOSD than in MS (22). High AQP4 expressing regions such as diencephalon (hypothalamus and thalamus) or dorsal midbrain (area postrema) are commonly affected in NMOSD (26). In MS, more supratentorial lesions are typically found in cortical, juxtacortical, or periventricular areas (27).

Contrast (Gadolinium) enhancement is a measure of BBB breakdown commonly found in MOGAD lesions. Within 4 weeks of the symptom onset, ON has enhancing pattern in most MOGAD (94%), NMO (100%) and MS (75%) patients (23). Myelitis, on the other hand, has an enhancement rate of 27–70% of MOGAD patients and around 75% of NMO or MS patients within 4 weeks of the symptom onset (24, 28).

Leptomeningeal enhancement, an indicator of leptomeningeal inflammation, was less frequently reported in MOGAD than in MS (29–31, 69). Gadde et al. reported the presence of LME in 33% (7/21) of a pediatric cohort. In comparison, Cobo-Calvo et al. reported in 6% (3/49) of their adult cohort (29, 30). Further studies are needed to determine if LME prevalence is higher (using 7T MRI) and if LME presence is associated with relapse activity in MOGAD. In MS, LME presence is reported as 79% with 7T MRI, while with lower field (1.5 or 3T MRI), prevalence is 21% (31). In NMOSD, it is also infrequent (6%) (31).

Central vein sign, defined as lesions with central vein identifiable by MRI, is commonly seen in MS (>40%), while their frequency is much lower in MOGAD (≈10%) or NMOSD (< 10%) (22).

Slowly expanding lesions are not present in MOGAD, while present in MS (25, 32). Paramagnetic rim lesions (PRL) associated with iron-laden microglia and macrophages, are present in MS; however, they have not been investigated in MOGAD (32–34).

### Histopathological findings

White matter lesions in MOGAD exhibit a confluent pattern around small veins, while in MS, focal lesions form around larger veins detectable by MRI (central vein sign) (50). Chronic active or slowly expanding lesions and iron rim lesions are absent in MOGAD while present in MS (50).

Cortical lesions in MOGAD also have perivenous confluent patterns and intracortical demyelination (50). In MS, subpial demyelination underneath the meningeal inflammation is common (51, 52). There are also ectopic lymphoid follicles found in MS, which are important aspect of chronic and progressive inflammation. In NMOSD, there is no cortical demyelination, while there is a neuronal loss in cortical layers II-IV (53, 54).

The dominant T cell type is CD4 in MOGAD, whereas CD8 is in MS (50, 55, 56). Astrocytes are spared in MOGAD, while pronouncedly decreased in NMOSD, and activated and proinflammatory in MS (49, 50, 58). Differential astrocyte involvement in these three diseases is also supported by GFAP levels in serum and CSF (59–61). GFAP-CSF levels are increased in the NMOSD patients but not in MOGAD compared to HC (60). In MS, progressive patients have higher GFAP both in serum and CSF compared to RRMS and HC (59). Conversely, oligodendrocytes are variably lost in all three diseases, while in MOGAD, progenitor cells are preserved as they do not yet express MOG (49, 50, 58, 62).

### Laboratory findings

Oligoclonal band presence is low in MOGAD (5–13%), similar to NMOSD (10–16%) (30, 41–43, 45, 46). In MS, however, OCB positivity is found in the majority of the cases (95%) (44).

CSF pleocytosis in MOGAD is quite common during relapses and even higher in the spinal cord (85%) or brain/brainstem (60%) involvement compared to optic neuritis (34%) (42). In NMOSD, a similar trend is present, with CSF pleocytosis in 24% of optic neuritis and 65% of myelitis (45). In MS, 50% of the patients have pleocytosis during relapses (47).

Increased albumin CSF/serum ratio (QAlb), a measure of blood-CSF barrier dysfunction, is also a feature of MOGAD. In two separate studies, almost one-third of MOGAD patients (32 and 32.4%) had increased albumin CSF/serum ratio (QAlb) (10, 46). This ratio was even higher in patients with a history of a spinal cord, brain, or brainstem involvement (10/21; 47.6%) (10). Elevated QAlb values are reported at a similar frequency with MS (29.5%, *n* = 606) (70). On the other hand, QAlb levels are reported variably for NMOSD (increased in up to 50–80% of patients), which implies implying blood-CSF barrier dysfunction in MOGAD may not be as severe as it is in NMOSD (45, 46, 71).

**Figure 2:**
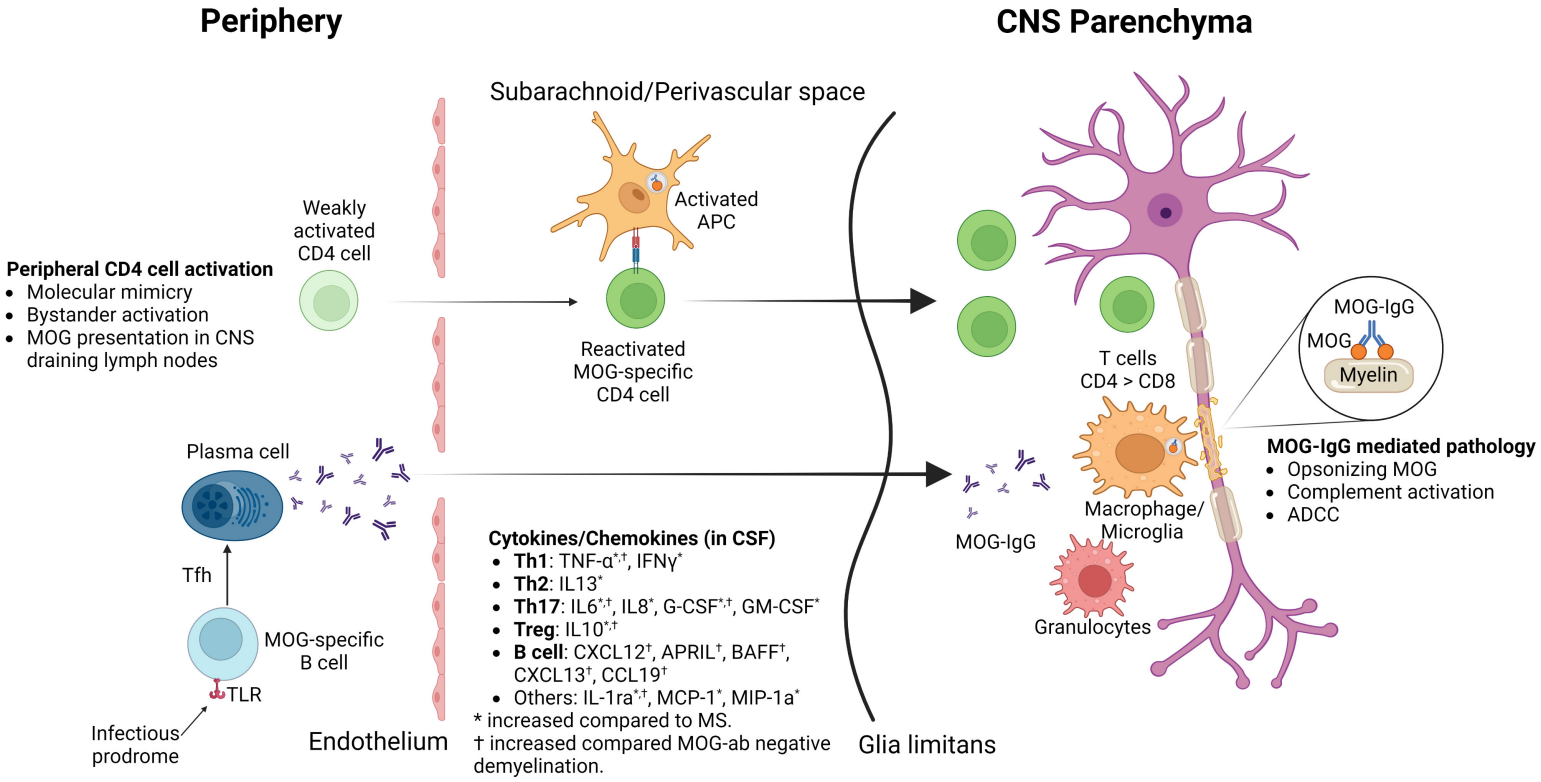
Pathophysiological actors in MOGAD.

CSF cytokine/chemokine profile of MOGAD shows increased proinflammatory cytokines (Figure 2, created with BioRender.com), including Th1 (TNF-α, IFNγ), Th2 (IL13), Th17 (IL6, IL8, G-CSF, GM-CSF), Treg (IL10) and B cell (CXCL12, APRIL, BAFF, CXCL13, CCL19) related and other (IL-1ra, MCP-1, MIP-1a) cytokines/chemokines (72, 73).

## Autoimmune etiology of MOGAD

The prevailing concept for autoimmunity in MOGAD is the outside-in model, where autoantibodies and activated immune cells in the peripheral blood cross the blood-brain barrier at the time of attack/relapse (Figure 2) (41, 74).

The central tolerance toward MOG may not be well developed in the thymus, preventing the elimination of MOG reactive T cells by negative selection (1, 75, 76). In the thymus, the expression of a self-antigen in the epithelial cells eliminates lymphocytes with a strong affinity to a self-antigen (central tolerance) and also allows some self-reactive T cells to develop into Tregs (peripheral tolerance) (77). MOG expression in the human thymus is variably reported. In two studies analyzing the thymus, MOG RNA was not detected, while in another study, MOG was detected in isolated medullary thymic epithelial cells (78–81). There is no protein-level study assessing MOG expression in human thymic tissue. Even if MOG expression in the thymus is low or present, central tolerance is not a perfect process, and peripheral tolerance mechanisms are needed to suppress self-reactive lymphocytes. Peripheral tolerance mechanisms include anergy or apoptosis of self-reactive T cells through the absence of costimulatory molecules or the presence of inhibitory molecules (such as PD1 or CTLA) and regulatory T cells (77). From MOG-induced EAE models and human MOGAD, we know that tolerance against MOG could be disrupted.

MOG reactive T cells, present due to a compromised tolerance against MOG, could be activated upon antigen-specific or non-specifically through mechanisms such as MOG peptide presentation, molecular mimicry, or bystander activation. MOG peptides could be present in the periphery, such as in cervical lymph nodes draining CNS, or rarely in a tumor expression MOG (82, 83). MOG antibodies, on the other hand, could facilitate recognition of trace amounts of MOG present in the periphery leading to T cell activation (84). Infections could cause bystander activation and molecular mimicry. Milk protein Butyrophilin and small Hepatitis B surface antigen are reported to have cross-immunoreactivity with MOG; however, pathophysiological consequences of these molecular mimicries have not been established (85, 86).

### Immunogenetics

Genetic risk factors associated with the autoimmune etiology of MOGAD are not widely explored. HLA genotyping studies did not identify a significant allele in two Dutch and UK cohorts (87, 88). However, a study in the Chinese Han cohort observed that DQB1^*^05:02 and DRB1^*^16:02 alleles and DQB1^*^05:02-DRB1^*^16:02 haplotype were more frequent in pediatric-onset MOGAD patients and DQB1^*^05:02-DRB1^*^16:02 haplotype was associated with higher initial EDSS and relapse risk (89).

### Role of infections in MOGAD

An infectious prodrome is commonly reported in MOGAD patients (37.5 to 70%) (7–10). An infectious prodrome could stimulate the underlying autoimmune processes by bystander activation, molecular mimicry, and epitope spreading. Infections could also disrupt peripheral tolerance by increasing costimulatory molecules and MHC-II expression on antigen-presenting cells (APCs) (90). This would lead to increased avidity of the interaction between APCs and self-reactive CD4+ T cells, which would otherwise go to anergy or apoptosis due to weak T cell stimulation.

Bystander activation of self-reactive B and T cells is one mechanism that could explain infectious prodrome commonly preceding attacks. In fact, pro-inflammatory cytokines that increase during infections, such as IL6 and TNFα, are also found to increase in CSF of MOGAD patients (38, 72, 73). In addition, Toll-Like receptor (TLR) activation caused by viruses could convert MOG-specific B cells into MOG-ab secreting plasmablasts (91). Separately, infections could directly or indirectly affect BBB as well (92, 93).

Since the COVID-19 breakout, multiple case studies reported COVID-19 infection preceding MOGAD onset (94, 95). The median time between COVID-19 to MOGAD diagnosis was 6 days (range: −7 to 45 days), while in a few cases, MOGAD diagnosis preceded or was concomitant with COVID-19 (94, 95). Further studies will show if the MOGAD incidence rate has increased after the pandemic.

### Anti-MOG IgG

The serum level of MOG IgG is an essential diagnostic and clinical biomarker. There is no consensus cut-off value for a diagnostic titer, which changes from center to center (35). Autoantibodies detected in MOGAD patients are of IgG1 type (96). MOG IgM levels do not correlate with anti-MOG IgG levels and could provide false positive results (96, 97). Persistent MOG-IgG positivity is associated with increased relapse risk (9). In monophasic MOGAD, MOG-IgG titers decrease over time, whereas in relapsing MOGAD, MOG-IgG titers tend to stay high (98).

Paired serum and CSF MOG-IgG positivity is found in more than half (56%) of the MOGAD patients (99). Some MOGAD patients are MOG-IgG seronegative and CSF positive, and CSF-restricted MOG-IgG may not always coexist with OCB positivity or elevated IgG index in the CSF (71). Therefore, in a strong clinical context (such as seronegative NMOSD or ADEM), CSF testing could help with MOGAD diagnosis (99, 100). Furthermore, CSF MOG-IgG positivity is associated with worse outcomes (99).

Detection of MOG-IgG antibodies in the serum by a live cell-based assay is the gold standard for diagnosis (35). Patient serum samples are incubated with live HEK293 cells expressing full-length MOG protein on their membrane, followed by secondary staining with anti-human IgG (H+L or Fc) or IgG1 (Fc) secondary antibodies. The analysis is done either by immune fluorescence microscopy or flow cytometry (35, 36).

Antibody response to MOG could be monoclonal or polyclonal. Mayer et al. tested the human MOG IgG binding pattern for seven different mutant human MOG proteins and mouse MOG proteins (101). They found that half of the patients showed decreased binding only to P42S (Proline to Serine) mutant, whereas about a third of the patients showed decreased binding to multiple mutants. These results indicated that an epitope containing P42 (proline at position 42) is the primary target of MOG IgG in half of the patients, and many patients have a polyclonal antibody response. Interestingly, immunoreactive epitopes are temporally stable, and there is no evidence of intramolecular epitope spreading (96, 101).

Pathogenicity of patient-derived purified MOG-IgGs was shown by Spadaro et al. by intrathecally injecting human MOG IgGs in an adoptive transfer EAE model (induced by MBP or MOG-specific T cells transferred to Lewis rats) (102). In this experiment MOG-antibodies were not pathogenic alone, and provided a second hit when interacted with T cells. When coupled with MBP-specific T cells, which are alone encephalitogenic and disrupt BBB, MOG abs mediated MS type II demyelination, characterized by complement (C9neo) and immunoglobulin deposition. On the other hand, when coupled with MOG-specific T cells, which do not induce clinical disease by themselves, MOG abs enhanced T cell recruitment and activation.

The source of the MOG abs in the CNS is mostly peripheral, although sometimes intrathecal production might be possible (41, 71, 74, 100). Through an impaired BBB (provided by activated T cells, infections, coexisting autoantibodies etc.), these MOG antibodies could enter the perivascular spaces and CNS, where they could contribute to disease pathology.

Suggested mechanisms for MOG antibody pathogenicity include opsonization of MOG, complement activation, antibody-dependent cellular cytotoxicity (ADCC), and anti-MOG ab-induced intracellular signaling cascade (3, 50, 84, 103, 104).

MOG-IgG opsonizes MOG and could activate myeloid antigen-presenting cells (APC) through Fc receptor binding (84, 104). These activated APCs can further stimulate MOG-specific T cells in the periphery or in perivascular spaces in the CNS (56).

The role of complement activation in MOGAD is still debated. IgG1 is a complement-fixing subtype, and there is evidence of C9neo deposition in the MOGAD histopathology (50). Furthermore, oligodendrocytes express relatively less surface complement regulatory proteins such as CR1, MCP, and HRF, making them more vulnerable to complement activation (106). In a multinational cohort study, serum-activated complement proteins (C3a, C5a, and Bb) were elevated in MOGAD patients compared to control groups (107). However, there was no correlation between activated complement protein levels in the serum and the clinical presentation (relapsing vs. monophasic or ADEM vs. ON vs. TM). On the other hand, complement activation in MOGAD is to a lesser extent, compared to AQP4 NMO (108). This might be because MOG-IgG has a bivalent binding pattern (both Fab subunits should bind to MOG), whereas AQP4-IgG has a monovalent binding pattern, which activates complement more efficiently (103, 108–111). This also indicates that anti-complement therapy may not be as successful as in NMO for MOGAD.

Another pathogenic mechanism is ADCC. Brilot et al. showed that MOG IgG binding to MOG induces Natural Killer cell-mediated killing of MOG-expressing cells *in vitro* (104).

MOG abs also have a direct downstream effect on oligodendrocytes (3). When Mog ab binds alone, it activates MAPK and AKT survival pathways and increases intracellular calcium levels, whereas cross-linking of the MOG abs leads to the activation of stress-related pathways and reduced cytoskeletal integrity.

### B cells

B cells are part of the disease pathogenesis through the production of MOG antibodies; however, there is much to discover about their contribution to the MOGAD. For example, anti-CD20 therapy is relatively ineffective in most patients. In a large cohort, rituximab decreased the relapse rate by 37%, and only 33% of patients remained relapse-free after 2 years (105). Failure of B cell depleting therapy suggests alternative pathogenic mechanisms in these patients, which could be used for enhanced T cell activation and MOG ab production. Serum MOG ab levels and circulating MOG-specific B cells did not correlate in MOGAD patients, raising the possibility of different MOG ab sources (for example, CD20- plasma cells) (91).

CXCR4 expression is increased in CD19+ B cells in PBMCs from MOGAD patients (112). Interestingly, CXCL12 (also called stromal cell-derived factor 1), a ligand of CXCR4, is also found increased in the CSF (compared to MOG ab- demyelination) and serum (compared to MS) of MOGAD patients (73, 113). As, CXCL12/CXCR4 axis is related with chemotaxis, increased CXCL12 could contribute immune cell infiltration (such as T, B, and monocytes) in the MOGAD (114). B cells are present in the lesions of MOGAD, although fewer than T cells (56). However, the source of MOG-IgGs is presumably the periphery, except in some patients with intrathecal production or CSF-restricted positivity (41, 50, 56, 71, 74, 100). Therefore, it is not certain if B cells cross BBB during MOGAD relapses and contribute to lesion formation within the CNS.

Altered regulatory B cells in MOGAD are reported in a study (115). Decreased Breg/Bmem ratio and decreased IL10+ CD19+ cell frequency are accompanied by increased circulating follicular T helper cells in MOGAD (115).

### T cells

T cells play a key role in MOGAD pathogenesis. First, MOG abs are IgG1 phenotype, so follicular T helper cells are essential for MOG-specific B cell class switching (96). Second, MOG abs are not pathogenic alone unless they are coupled with MOG-specific or encephalitogenic T cells (102). CD4+ T cells are the dominant inflammatory cell type in the lesions and have an essential role in disrupting BBB and creating a proinflammatory environment (50, 116).

MOG-specific T cells are first activated peripherally (90). Due to inadequate tolerance toward MOG, MOG-specific T cells could be present in the blood. Infections, molecular mimicry, and MOG peptide presentation could facilitate the activation of self-reactive T cells. Then, peripherally activated CD4+ T cells cross BBB and are reactivated by the MOG-laden APCs in the perivascular or subarachnoid spaces (56). This reactivation is followed by endothelial and microglial activation, allowing more T cells and autoantibodies to enter the perivascular space (90, 116).

The strength of T cell reactivation and chemokines in the CNS can facilitate parenchymal infiltration of the CD4+ T cells (90, 117, 118). Anti-MOG abs help APCs (such as macrophages) to present native MOG protein to T effector cells and enhance the reactivation of effector T cells in the CNS (84, 119). T cells could be reactive to different epitopes of MOG protein. Most immunogenic MOG epitopes were previously determined in EAE models and tested MS patients (120, 121). These include p35–55, p119–130, p181–195, and p186–200. The following study tested nine different MOG peptides (p1–20, p35–55, p64–80, p81–96, p99–107, p119–130, p181–195, p186–200, and p205–214) in MOGAD, AQP4+ NMO, MS, and HC, but didn't find a difference of MOG-specific T cells between any groups based on CFSE assay (122). This could be explained by the lack of central tolerance and, therefore, the presence of MOG reactive T cells even in HC. Another possibility is that native full MOG protein is required for an optimal T-cell response.

Th17 responses are higher in MOGAD patients. Our lab used different MOG peptides for *in vitro* stimulation and showed MOGAD patients had more IL17+ and IL17+IFNγ+ (double positive) central memory cells than healthy controls (123). When relapse and remission MOGAD samples were compared, there was an increased proportion of IL17+, IFNγ+, and IL17+IFNγ+ CMCs after stimulation with several individual MOG peptides in MOGAD patients at the time of relapse (123). Later, Horellou et al. stimulated PBMCs with recombinant full-length MOG protein (rhMOG) and observed increased IL17+ CD4 T cells in non-relapsing (monophasic) MOGAD patients upon rhMOG stimulation, but not in relapsing MOGAD or MS (124).

Tregs play an important role in peripheral immune tolerance. Horellou et al. reported an increased CD4+ Foxp3+ Treg population in the non-relapsing MOGAD group and decreased CD45RA-Foxp3+ Treg population in MOGAD relapsing group upon rhMOG stimulation (124). They hypothesized that this opposite response to MOG stimulation could contribute to the relapse mechanism. Besides, we don't know if Tregs are functional in MOGAD; however, inflammatory milieu (high IL6 and TNF) in MOGAD could render Tregs ineffective with suppressing (72, 113, 125).

### Innate immune cells

Macrophage (CD68+) and granulocyte (hematoxylin-eosin) infiltration is reported in MOGAD lesions, especially in the perivascular demyelinating areas (50, 56). The presence of phagocytic macrophages indicates active demyelination, and MOG-laden macrophages could further activate T cells in the perivascular spaces (56).

IL-1β and IL-12p70, monocyte or dendritic cell-related cytokines, are increased in the serum of MOGAD patients (126). IL-1β levels were highest in the acute stage and lower in the chronic phase, indicating monocyte/macrophages play a role in the acute demyelinating stage (126). IL-1β also affects the permeability of endothelial cells *in vitro* human BBB model (127).

In a recent study of three patients (1 MOGAD, 1 RRMS, and 1 Healthy control), single-cell RNA sequencing of PBMCs revealed changes in monocyte signature in MOGAD compared to RRMS or HC, however, a larger, age and sex-matched cohort is needed to confirm these findings (112).

The increased neutrophil-to-lymphocyte ratio (NLR) in serum is also reported in MOGAD, with relapse samples having higher NLR values than the remission sample (128). This biomarker requires caution as many confounding factors, such as hospitalization and steroid treatment, could affect NLR.

### Coexisting antibodies

Epitope spreading is not established in MOGAD, however, coexisting antibodies have been investigated. Kunchok et al. tested 17 neuronal IgGs in the CSF and serum of pediatric and adult MOGAD patients and found NMDA-R-IgG is the most frequent coexisting autoantibody (4% in children and 7% in adults) (129). On the other hand, anti-Aqp4 IgG and anti-MOG IgG rarely coexist (0.06%) (130).

Serum-IgG from demyelinating animal models or patients could also affect BBB permeability by affecting pericyte function and lymphocyte adhesion molecule (LAM) expression on endothelial cells (131, 132). Interestingly, anti-GRP78 antibodies were reported to be commonly present in acute MOGAD patients (10/15, 67%) and caused BBB dysfunction through increased LAM expression in endothelial cells and NF-κB activation (133).

In summary (Figure 2), MOGAD pathophysiology is driven by acute attacks during which T cells and MOG antibodies cross BBB. Initial activation of T cells in the periphery is followed by reactivation in the subarachnoid/perivascular spaces by MOG-laden APCs and inflammatory CSF milieu, which enables T cells to infiltrate CNS parenchyma. CD4 T cells, unlike CD8 in MS, are the dominant T cell type found in lesion histology. Granulocytes, macrophages/microglia, and activated complement are also found in the lesions, which could contribute to demyelination during acute relapses. MOG antibodies potentially contribute to pathology by opsonizing MOG, complement activation, and antibody-dependent cellular cytotoxicity. Stimulation of peripheral MOG-specific B cells through TLR stimulation or T follicular helper cells might help B cells differentiate into MOG antibody-producing plasma cells in the peripheral blood.

## Lesion topography in MOGAD

Different clinical presentations and lesion distribution seen in MOGAD is an intriguing topic. What is the difference between MOG-IgG-related optic neuritis, transverse myelitis, and tumefactive lesions in ADEM?

Higher antibody levels in serum can favor spinal cord involvement against the optic nerve. Jarius et al. reported that MOG antibody titers are higher during relapses compared to remission, and relapses involving myelitis have higher titers of MOG antibodies compared to isolated optic neuritis (48).

Expression levels of MOG protein across different parts of the CNS could contribute to differential lesion involvement. For example, Bettelli et al. reported that 2D2 TCR transgenic mice, which have MOG-specific T cell receptors, developed spontaneous autoimmune optic neuritis but not spinal cord lesions, which they associated with higher MOG expression in the optic nerve compared to the spinal cord (134). Comparative expression of human MOG protein in optic nerve and CNS has not been reported, although MOG expression in different parts of the human brain and spinal cord has been variably reported in the human protein atlas (80, 135). Consequently, we do not know if the most common lesion location (optic nerve in adults, brain in children) relates to MOG expression level in those tissues.

The Th17:Th1 ratio could affect lesion topography. PBMCs from MS patients were stimulated with MOG or MBP proteins, and higher Th17:Th1 upon MOG (recombinant human) stimulation was associated with spinal cord involvement (136). Interestingly, epitope-specific T cell functional avidity help determine Th17:Th1 ratio in MOG peptide-induced EAE model (C3HeB/Fej mice, p35–55, p79–90, p97–114 MOG peptides) (137). So, maybe also in MOGAD, different epitope/TCR reactivity in T helper cells brings differential Th17:Th1 balance, affecting the lesion topography.

The quantitative relationship between inflammatory T cells and autoimmune antibodies could explain the size and structure difference of lesions in MOGAD (50, 138). Lassmann et al. tested this hypothesis with encephalitogenic T cells (MBP-specific) and MOG antibodies in an EAE model. Higher numbers of T cells in the presence of MOG abs induced an ADEM-like phenotype with ubiquitous perivenous demyelination all over the brain stem and spinal cord and partly in the brain. In contrast, low T cell and high MOG ab titer induced focal demyelinating plaques like in MS.

## Biomarkers and therapeutic mechanisms

Potential biomarkers investigated with MOGAD are listed below (Table 2). First, MOG-IgG is both a diagnostical and prognosis biomarker. High titers are needed for MOGAD diagnosis, and seronegative conversion is associated with decreased relapse risk (9). Furthermore, relapsing MOGAD patients have higher MOG-IgG titer at remission compared to monophasic MOGAD (139). Treatments such as IVIG, Plasmapheresis, or FcRn blockers target pathogenic antibodies in the blood, including MOG-IgG. IVIG has been used commonly for maintenance treatment in MOGAD, and potential therapeutic mechanisms include increasing clearance of pathogenic antibodies by saturating neonatal FcR (FcRN) and blockade of activating FcγRs (144). Similarly, anti-FcRn antibodies also increase the pathogenic ab clearance, and currently, rozanolixizumab, an anti-FcRn agent, is in phase 3 clinical trial for relapse prevention in MOGAD (NCT05063162).

**Table 2:** Biomarkers investigated with MOGAD.

**Potential biomarkers**		**MOGAD disease activity**	**Significance/implications**	**Therapeutic mechanism**
MOG-IgG titer (9, 98, 139)	Serum	High titers required for diagnosis	Seronegative conversion indicates decreased relapse risk	FcRn blockers, IVIG, Plasmapheresis
NfL (140, 141)	Serum	Increased mostly in the first attack, then stay stable throughout the course	Significant axonal damage happens mostly in the first attack	
MBP (60)	CSF	Increased	Marker of demyelination	
GFAP (60, 141)	Serum	Stay stable during relapses	Spared astrocytes	
Tau (141)	Serum	Increased during relapses	Synthesized in axons and oligodendrocytes	
IL6 (72, 73, 113, 142)	CSF, serum	Increased in the CSF during relapses	Increased STAT3 activation could cause increased Th17	IL6 receptor blockers
			Impair BBB	
TNFα (42, 43, 143)	CSF	Increased in the CSF during relapses	May affect BBB through increased cell adhesion molecule expression (such as ICAM-1 and VCAM-1)	
A20/TNFAIP3 (123)	Serum	Decreased in the serum during relapses (individual level)	Increased NFKB activation	Steroids (increase)
	Intracellular		Steroid increase A20 expression in T cells	
N/L ratio blood (128)	Blood	Increased ratio during relapses	Could help differentiating relapse from pseudo-relapse	

Serum NfL (sNfL) level is a biomarker for axonal damage and correlates with relapse activity in MS. A recent study evaluated longitudinal sNfL values from 18 MOGAD patients and found that median sNfL levels at the onset are higher compared to age-matched HC (140). Most follow-up sNfL values stayed stable or decreased over time, including relapse serum samples of 6 patients. In the following study, sNFL levels were not different between relapse and remission MOGAD samples (141). This observation supports that significant axonal damage happens in the first clinical attack (30, 140).

Increased MBP (CSF) and Tau levels (serum) in MOGAD suggest that there is myelin and oligodendrocyte damage (60, 141). Increased Tau levels in the relapse, but not sNFL, may support that the source of Tau could be damaged oligodendrocyte processes, not axons (141).

Serum GFAP levels are stable during relapses in MOGAD (141). This finding is compatible with the spared astrocytes seen in the biopsy. In NMOSD, astrocyte damage is pronounced and serum GFAP levels are increased during relapses (141). In MS, increased GFAP is seen with progressive disease (145).

IL6 and TNFα levels are increased during relapses in the CSF of MOGAD patients (43, 44). Recently, increased serum IL6 levels are also reported in MOGAD (113). Increased IL6 is associated with Th17 differentiation through IL6-STAT3 pathway and impaired BBB (142). Recently, a multinational study showed that IL6 receptor blockade with tocilizumab is therapeutically effective and safe in MOGAD and NMOSD (146). Increased TNFα, on the other hand, could also affect BBB permeability through leukocyte adhesion molecule expression such as ICAM-1 and VCAM-1 (143). Although, steroids may decrease TNFα synthesis, we do not have information about targeted therapies such as TNF receptor blocking, while few cases in the literature are diagnosed with MOGAD while on anti-TNF treatment due to preexisting other autoimmune diseases (147, 148).

We recently reported that A20, a negative regulator of the NF-κB pathway, is decreased in the serum during relapses on an individual level (123). Interestingly, steroids increase A20 expression in CD4 T cells. As well known, MOGAD patients are quite steroid-responsive, and it is difficult to taper steroids following relapses (149). In addition to inhibiting the NF-κB pathway through A20, steroids improve BBB and decrease activation of T cells, thereby minimizing the migration of lymphocytes into the brain (150–152).

NLR is increased in MOGAD patients during relapse, supporting high inflammatory activity in the periphery (128). However, this biomarker should be utilized carefully as factors such as hospitalization and steroid treatment affect NLR.

Micro RNAs (miRNAs) have not been fully explored in MOGAD. Only, a study measured miR-17, miR-18a, miR-20a, and miR-92a-1 in PBMCs (12 MOGAD, 12 HC) *via* qPCR and found them increased in MOGAD (153). In the EAE model, miRNA-17 increased Th17 and miRNA-20a decreased Treg fraction (153, 154). Further miRNA screening studies (serum or PBMCs) are needed to confirm these miRNAs and identify other miRNAs.

Developing a peripheral immune tolerance against MOG is a promising therapeutic method. For example, intradermal MOG vaccination increased MOG-specific Tregs and improved clinical outcomes in the macaque EAE model (rhMOG induced) (155). More recently, Ugur Sahin et al. used an mRNA vaccine to induce tolerance in a mouse EAE model (C57BL/6 mice, MOG p35-55 induced) (156). This mRNA vaccine provided a self-antigen presentation in a non-inflammatory context, expanded MOG-specific Tregs, and increased expression of inhibitory molecules such as PD-1 and CTLA4. Furthermore, when the mRNA vaccine was administered on days 7 and 10 after immunization with MOG p35–55, the development of EAE was prevented, and administration after the EAE onset alleviated the symptoms.

## Discussion

Frequent infectious prodrome and high IL6/IL17 associated cytokine-chemokine signature are important components of MOGAD (113). Within this inflammatory milieu in the peripheral blood, MOG-specific T cells, especially CD4+, are activated through bystander activation, molecular mimicry, or maybe MOG peptide presentation in CNS-draining lymph nodes. Peripherally activated T cells further open blood-brain barrier through reactivation in the perivascular spaces, allowing autoantibodies, complement, and more immune cells to enter to perivascular spaces and CNS (90, 116). In addition to CD4+ T cells, granulocytes, macrophages/microglia, and activated complement are also found in the lesions contributing to demyelination (50, 56).

In addition to being diagnostical and prognostic biomarker, MOG antibodies potentially contribute to pathology by opsonizing MOG, complement activation, and antibody-dependent cellular cytotoxicity. MOG antibodies enhance T cell-mediated inflammation in the CNS, however, MOG antibodies alone are not pathogenic (102). MOG-IgG is detected in the CSF of more than half of the MOGAD patients and associated with worse outcomes compared to CSF negative MOGAD patients (99). Since MOG antibodies require BBB dysfunction to enter CNS, a trigger involving T cell activation and BBB dysfunction is important for the onset.

BBB and blood-CSF barrier dysfunction in MOGAD is evidenced by contrast enhancing lesions and increased albumin quotient (32%) (10, 46). Increased pro-inflammatory cytokines (such as IL6, TNFα, IL-1β, MCP-1) could also contribute to BBB dysfunction (72, 73, 113). Available treatments such as plasmapheresis, IVIG (or FcRn blockers), steroids, and IL6 receptor blockers may affect BBB directly or indirectly through decreasing inflammatory cytokines and coexisting autoantibodies in the plasma.

Neuroinflammatory biomarkers (such as MBP, sNFL, GFAP, Tau) in MOGAD support that most axonal damage happens in the initial attack, as evidenced by increased sNFL (140, 141). Demyelination associated with myelin and oligodendrocyte damage is evidenced by increased MBP (CSF) and Tau levels (serum, during relapse) [51, 135].

Understanding the autoimmune etiology of MOGAD will help us to identify biomarkers, predict prognosis, and find targeted therapies. For example, therapeutic mechanisms targeting IL6, MOG-IgG (FcRn blocking or IVIG), or improving peripheral tolerance (Treg-inducing MOG-vaccine) are new avenues that will benefit our patients.

## Author contributions

The first draft of the manuscript was written by OC. All authors commented on previous versions of the manuscript, contributed to the manuscript conception and design, read, and approved the final manuscript.
